# Assessment of radiobiological metrics applied to patient‐specific QA process of VMAT prostate treatments

**DOI:** 10.1120/jacmp.v17i2.5783

**Published:** 2016-03-08

**Authors:** Francisco Clemente‐Gutiérrez, Consuelo Pérez‐Vara, María H. Clavo‐Herranz, Concepción López‐Carrizosa, José Pérez‐Regadera, Carmen Ibáñez‐Villoslada

**Affiliations:** ^1^ Sección de Radiofísica Servicio de Oncología Radioterápica Hospital Central de la Defensa “Gómez Ulla” Madrid Spain; ^2^ Servicio de Oncología Radioterápica Hospital Central de la Defensa “Gómez Ulla” Madrid Spain; ^3^ Servicio de Oncología Radioterápica Hospital Universitario Doce de Octubre Madrid Spain

**Keywords:** VMAT QA, pretreatment verifications, 3D dose reconstruction, radiobiological metrics

## Abstract

VMAT is a powerful technique to deliver hypofractionated prostate treatments. The lack of correlations between usual 2D pretreatment QA results and the clinical impact of possible mistakes has allowed the development of 3D verification systems. Dose determination on patient anatomy has provided clinical predictive capability to patient‐specific QA process. Dose‐volume metrics, as evaluation criteria, should be replaced or complemented by radiobiological indices. These metrics can be incorporated into individualized QA extracting the information for response parameters (gEUD, TCP, NTCP) from DVHs. The aim of this study is to assess the role of two 3D verification systems dealing with radiobiological metrics applied to a prostate VMAT QA program. Radiobiological calculations were performed for AAPM TG‐166 test cases. Maximum differences were 9.3% for gEUD, −1.3% for TCP, and 5.3% for NTCP calculations. Gamma tests and DVH‐based comparisons were carried out for both systems in order to assess their performance in 3D dose determination for prostate treatments (high‐, intermediate‐, and low‐risk, as well as prostate bed patients). Mean gamma passing rates for all structures were better than 92.0% and 99.1% for both 2%/2 mm and 3%/3 mm criteria. Maximum discrepancies were (2.4%±0.8%) and (6.2%±1.3%) for targets and normal tissues, respectively. Values for gEUD, TCP, and NTCP were extracted from TPS and compared to the results obtained with the two systems. Three models were used for TCP calculations (Poisson, sigmoidal, and Niemierko) and two models for NTCP determinations (LKB and Niemierko). The maximum mean difference for gEUD calculations was (4.7%±1.3%); for TCP, the maximum discrepancy was (−2.4%±1.1%); and NTCP comparisons led to a maximum deviation of (1.5%±0.5%). The potential usefulness of biological metrics in patient‐specific QA has been explored. Both systems have been successfully assessed as potential tools for evaluating the clinical outcome of a radiotherapy treatment in the scope of pretreatment QA.

PACS number(s): 87.56.Fc, 87.55.Qr, 87.55.dk, 87.55.dh, 87.10.Vg, 87.55.km, 87.53.Bn, 87.55.‐x, 87.56.‐v

## I. INTRODUCTION

Radiation therapy (RT) for prostate cancer has substantially evolved during the last years. Dose escalation improves disease control, at the expense of an increment in toxicity.^(1)^ Modulated techniques can reduce toxicity by optimizing treatment conformation.^(2)^ Hypofractionated schemes are suitable in prostate treatment because of the low α/β ratio for the prostate gland.^(3)^ Hypofractionated plans delivered with intensity‐modulated radiation therapy (IMRT) techniques lead to extended treatment times compared to traditional techniques. Volumetric‐modulated arc therapy (VMAT) has been developed due to rotational capabilities recently implemented in conventional linacs.^(4)^ Treatment times are noticeably reduced within this new paradigm, making VMAT a powerful tool for hypofractionated prostate treatments.^5^, ^6^, ^7^, ^8^, ^9^

VMAT, as one kind of IMRT technique, requires a detailed patient‐specific quality assurance (QA) program.^(9)^ This independent pretreatment QA process usually consists of comparing dose measurements acquired with phantoms/detectors of regular geometries with treatment planning system (TPS) calculations made under the same conditions.^10^, ^11^ Ion chambers are used to perform point measurements. Two‐dimensional (2D) (plane) dose distributions are measured with several systems: electronic portal imaging devices, films or 2D detector arrays. Tests involving gamma index passing rates are common in these comparisons.^12^, ^13^ Three‐dimensional (3D) verifications start with specifically developed solutions for volumetric techniques.^14^, ^15^ The lack of correlations between usual 2D pretreatment QA results and the clinical impact of possible mistakes has been established.^16^, ^17^ In a second step, based on previous conclusions, 3D verification systems are developed under the scope of determining dose on patient anatomy, providing clinical predictive capability to these systems. Solutions for redundant calculations on patient CT information or 3D dose reconstruction from measurements have already been developed.^(18)^ Three‐dimensional dose calculation and reconstruction have introduced DVH‐based metrics in QA process, allowing for dose‐volume information comparisons.

The quality of an RT plan has been traditionally judged by dose‐volume parameters rather than biological ones. However, dose‐volume criteria should be complemented by biological indices.^(19)^ Eventually, biological models should be routinely introduced and validated because these models have demonstrated their predictive ability in the evaluation of the treatment outcome. Although a whole replacement of standard DVH‐based metrics should not be recommended, the efforts may be addressed in order to validate outcome prediction models, overcoming the traditional evaluation metrics.^20^, ^21^, ^22^, ^23^ Radiobiological data and response parameters — such as generalized equivalent uniform dose (gEUD),^24^, ^25^ tumor control probability (TCP),^26^, ^27^, ^28^ or normal tissue complication probability (NTCP)^29^, ^30^, ^31^, ^32^, ^33^, ^34^, ^35^ — can be obtained from DVH information. Hence, radiobiological metrics can be incorporated into individualized pretreatment QA process. This paper assesses the role of two 3D dose verification systems dealing with radiobiological metrics applied to a VMAT prostate treatment QA program.

## II. MATERIALS AND METHODS

### A. Treatment unit and TPS

VMAT plans were generated with Monaco 3.1 (Elekta; Stockholm, Sweden). Treatments were delivered with a monoenergetic (6 MV) Synergy (Elekta) machine.

### B. 3D dose verification systems

Two 3D verification systems were assessed. Mobius3D software (Mobius Medical Systems, Houston, TX) provides an independent dose calculation engine for treatments generated by TPS. COMPASS (v. 3.1) (IBA Dosimetry, Schwarzenbruck, Germany) is capable of reconstructing dose on patient CT from measurements taken with an associated detector. In addition, it provides an independent and redundant dose verification of TPS calculations, as does Mobius3D.

Both require, as initial information, DICOM treatment plan information (CT images, RTPlan, RTStruct, and RTDose).

#### B.1 Mobius3D system description

The software uses stock reference values for common linear accelerators to model the beams. Mobius3D works with a collapsed cone convolution/superposition algorithm independently developed and updated from its original conception.^36^, ^37^, ^38^, ^39^, ^40^, ^41^ The algorithm is accelerated throughout graphic processing units (GPUs), increasing the calculation speed significantly compared to CPUs.

#### B.2 COMPASS system description

COMPASS consists of two different devices: the detector and associated software. The detector device is a 2D ion chamber array (${\text{MatriXX}}^{\text{Evolution}}$, IBA Dosimetry). It has 1020 ion chambers (0.08 cm^3^) covering an active area of $24.4\times 24.4\;{\text{cm}}^{2}$. This detector has already been evaluated for VMAT pretreatment QA.^(42)^ The MatriXX device must be placed in a holder attached to the head treatment unit in order to ensure a rigid rotation of the detector with the gantry. Source‐to‐detector distance is 100 cm. A buildup thickness of 2.5 cm was used with the previous arrangement. An angle sensor is attached to the gantry in order to associate each measured fluence with its detection angle; the sensor has an angular tolerance of ±0.6°. COMPASS software requires a beam modeling process fitting basic parameters, as expected for TPS. The model connects with a collapsed cone convolution/superposition algorithm that allows both calculating and reconstructing (from measurements) dose on patient CT. A commissioning process is required for the MatriXX device in the software. It consists of background (20 s) and pre‐irradiation (5 Gy or higher) measurements together with a square field $\left(10\times 10\;{\text{cm}}^{2}\right)$ acquisition that automatically corrects detector shifting and rotation. An absolute dose calibration with a known‐dose reference field is also required. Sampling time for each measurement was 250 ms.

### C. Dose‐response models

#### C.1 gEUD

For a nonuniform tumor dose distribution, equivalent uniform dose (EUD) is defined as the uniform dose that yields the same biological effect, if treatment is delivered over the same number of fractions as the nonuniform original dose distribution.^(24)^ Niemierko^(25)^ proposed a phenomenological expression to extend the previous concept to normal tissues, referred to as the generalized EUD (gEUD):

(1) $$gEUD={\left(\sum_{j}v_{j}d_{j}^{a}\right)}^{1/a}$$

where $v_{j}$ is the fractional tumor volume receiving a dose $d_{j}$, and *a* is a tissue‐specific parameter describing volume effect. For tumors, *a* takes negative values; for serial‐like structures, *a* takes large positive values; and for parallel‐likes structures, *a* takes values close to 1.

#### C.2 TCP

Tumor control probability (TCP) can be modeled as a Poisson distribution.^(26)^ If the number of initial clonogen cells is $N_{c}$ and the clonogen surviving fraction after irradiation with a single fraction is denoted by S, TCP can by written for a course of n fractions as:

(2) $$TCP=e^{-N_{c}S^{n}}$$

Assuming that the average number of surviving clonogenic cells is an exponential function of the dose, the characteristic sigmoid dose‐response curve is obtained. This simple assumption has been replaced by introducing the linear‐quadratic (LQ) model to obtain the surviving fraction^(27)^ as:

(3) $$S=e^{-\alpha d\left(1+\frac{d}{\alpha /\beta }\right)}$$

where *d* is the dose per fraction and α/β are usual parameters in the LQ model. For an inhomogeneous irradiation of dose $d_{j}$ in a fractional volume $v_{j}$, TCP can be calculated as^(28)^ (Poisson model for TCP):

(4) $$TCP=\prod_{j}\text{exp}\left\{-N_{c}v_{j}\text{exp}\left[-n\alpha d_{j}\left(1+\frac{d_{j}}{\alpha /\beta }\right)\right]\right\}$$

The number of clonogenic cells can be determined using some of the following relations:)^43^, ^44^, ^45^

(5) $$N_{c}=\text{ln}2\;\text{exp}\left[-D_{50}\alpha d_{j}\left(1+\frac{d_{j}}{\alpha /\beta }\right)\right]$$

(6) $$N_{c}=\frac{-\text{ln}2}{\text{ln}\left\{1-\text{exp}\left[-D_{50}\alpha d_{j}\left(1+\frac{d_{j}}{\alpha /\beta }\right)\right]\right\}}$$

where $D_{50}$ is the tumor dose required to obtain a TCP of 50%. In addition, an expression for TCP may be obtained if data of clonogenic cells are not available, in terms of sigmoidal dose response parameters as^43^, ^44^, ^46^ (sigmoidal model for TCP):

(7) $$TCP={\left(1/2\right)}^{\sum_{j}v_{j}\text{exp}\left[\frac{2\gamma_{50}}{\text{ln}2}\left(1-\frac{d_{j}}{D_{50}}\right)\right]}$$

The $\gamma_{50}$ parameter is the slope of dose response at TCP of 50%. Another possibility consists of using gEUD concept previously introduced, obtaining TCP as^(28)^ (Niemierko model for TCP):

(8) $$TCP=\frac{1}{1+{\left(D_{50}/gEUD\right)}^{4\gamma_{50}}}$$

#### C.3 NTCP

The Lyman model^(29)^ describes complication probabilities for uniformly irradiated organ volume. The characteristic sigmoid dose‐response curve is described by three parameters. Sigmoid curve dependence on dose is described by ${\text{TD}}_{50}$ and curve steepness by m. The magnitude of the volume effect is described by n parameter in a power‐law relationship between the tolerance dose and irradiated volume:

(9) $$TD(v)=TD(1)v^{-n}$$

where *TD(v)* is the tolerance dose for a given partial volume fraction *v*, and *TD(1)* is the tolerance dose for the full volume. For an inhomogeneous irradiation, the Lyman model can be completed with an algorithm to convert a heterogeneous dose distribution into a uniform organ irradiation resulting in the same NTCP. The effective volume method^(31)^ is most commonly used to complement the Lyman model, resulting in a combined formalism named the Lyman‐Kutcher‐Burman (LKB) model. NTCP can be calculated, for an inhomogeneous irradiation of dose $d_{j}$ in a fractional volume $v_{j}$, as:^31^, ^32^

(10) $$NTCP=\frac{1}{\sqrt{2\pi }}\int_{\infty }^{t}e^{-u^{2}/2}du$$

where *t* is

(11) $$t=\frac{D_{eff}-TD_{50}}{mTD_{50}}$$

with D_eff_

(12) $$D_{eff}={\left(\sum_{j}v_{j}d_{j}^{1/n}\right)}^{n}$$

Another possibility entails using the concept of gEUD concept previously introduced and obtaining NTCP in the Niemierko model as:^32^, ^34^

(13) $$NTCP=\frac{1}{1+{\left(TD_{50}/gEUD\right)}^{4\gamma_{50}}}$$

### D. Prostate treatments

Prostate treatments were planned and delivered with a single arc. VMAT technique was applied to all prostate cases treated with external beam therapy: treatment for usual staging (high‐, intermediate‐ and low‐risk) and radiotherapy after prostatectomy. Prostate gland, seminal vesicles, and pelvic lymph nodes were treated in high‐risk patients; prostate gland and seminal vesicles were the targets for intermediate‐risk cases; and prostate volume was the single target for low‐risk staging. A simultaneous integrated boost (SIB) technique was used with two or three target volumes. A moderate hypofractionation scheme was applied for the previous three staging levels. The prescription doses were 70 Gy to prostate gland, 56 Gy to seminal vesicles, and 50.4 Gy to pelvic lymph nodes, delivered in 28 fractions. For prostate bed treatments after prostatectomy, the prescription dose was 74 Gy delivered in 37 fractions. Contoured organs at risk (OARs) were usually rectum, bladder, and femoral heads. For each usual staging, 25 prostate cases were analyzed, together with 25 cases of prostate bed treatments, for a total of 100 analyzed treatments.

### E. Verifications of biological metrics for both systems with TG‐166 benchmark phantom and test cases

In order to test the capabilities of both systems for radiobiological calculations, tests taken from the AAPM TG‐16621 report were performed.

#### E.1 Benchmark phantom test

**Figure 1:**
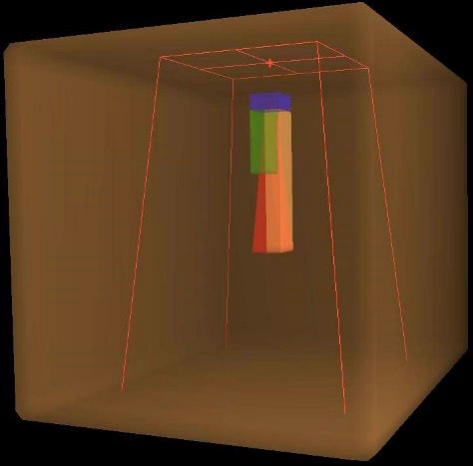
Benchmark phantom test case from the AAPM TG‐166 report,^(21)^ with four simple structures (three rectangular, one triangular) and the edge of a single 6 MV, 100 cm source‐to‐surface distance, $20\times 20਀{\text{cm}}^{2}$ photon beam.

Benchmark phantom consists of a large cubical phantom with four simple structures (three rectangular, one triangular) created inside the phantom^(21)^ (Fig. 1). A single 6 MV, 100 cm source‐to‐surface distance, $20\times 20਀{\text{cm}}^{2}$ photon beam was calculated in the TPS, with a prescribed dose of 72 Gy in 40 fractions to a point at 6 cm depth along the central axis. Dose imparted over benchmark phantom structures was determined by Mobius3D (M3D), COMPASS calculation (CC), and reconstruction (CR) modules; then, gEUD, TCP, and NTCP were calculated and compared among TPS, M3D, CC, and CR from the previous models. An in‐house developed software was used to perform previous calculations. The software reads the DVH information from the different systems and applies Eqs. (1), (4), (5), (6), (7), (8), (10), (11), (12), and (13) in order to obtain gEUD, TCP, and NTCP values.

#### E.2 Representative test cases

Treatment plans for three representative test cases (head and neck [H&N], prostate, and brain) were also calculated in the TPS according to the volumes and dose prescriptions defined in the report. gEUD, TCP, and NTCP values were determined for TPS plans and compared to those extracted by M3D, CC, and CR for the same treatments.

### F. Evaluation of prostate treatments with classical (gamma) and DVH‐based dose‐volume metrics

In order to assess correct performance in 3D dose calculation and reconstruction processes, traditional gamma tests and DVH‐based comparisons were carried out for both systems. Resorting to classical metrics, comparisons with TPS by means of global gamma passing rates for all structures were reported with two criteria (2%/2 mm and 3%/3 mm, global normalization to maximum, with a low‐dose threshold at 10% of global maximum). In addition, 3D dose evaluation was performed by comparing all prostate plans generated by the TPS and those determined by M3D, CC, and CR. Representative dosimetric parameters were obtained from DVHs. ICRU recommendations for recording and reporting IMRT treatments^(20)^ were used to extract evaluation parameters for PTVs ($D_{98},D_{2},D_{50}$, D_mean_). Maximum and mean doses were extracted for OARs. For normal tissue, depending on the case, classical^(47)^ and recently reviewed dose constraints (QUANTEC)^(48)^ were also reported.

### G. Introducing biological metrics in patient‐specific QA for prostate treatments

gEUD, TCP, and NTCP were extracted from TPS and compared to those values obtained with M3D, CC, and CR for all the models discussed above. These calculations were performed with the same in‐house software previously defined. In order to support the introduction of radiobiological metrics, the correlation between biological indices and differences in DVH parameters was studied.

### H. Radiobiological parameters

Parameters used for tumor calculations (gEUD and TCP) for TG‐166 test cases and prostate treatments are summarized in Table 1. Values for a parameter were extracted from the TG‐166 report^(21)^ (value for benchmark phantom case was also taken as −10). Selected α value was $0.1\;{\text{Gy}}^{-1}$.^(49)^ Selected α/β values were 10 Gy for TG‐116 cases, with the exception of the prostate case, where 3 Gy was selected,^(45)^ therefore taking values recommended in the report. For analyzed prostate treatments, α/β was 1.5 Gy, which is customary in our institution.^50^, ^51^, ^52^, ^53^ $D_{50}$ and $\gamma_{50}$ values for benchmark phantom case were taken from the TG‐166 report. Values for the remaining TG‐116 cases were also extracted from the study by Okunieff et al.^(45)^ For the analyses of high‐, intermediate‐, and low‐risk prostate treatments, $D_{50}$ and $\gamma_{50}$ values were extracted from studies by Cheung et al.^54^, ^55^ For prostate bed treatments, parameters were taken from the study by King et al.,^(56)^ using the relationship between absolute and relative slope at D_50_.^(45)^ In addition, TCP values for the prostate cases under analysis were calculated with the values reported by Okunieff et al.^(45)^ and Levegrun et al.^(57)^

**Table 1:** Selected radiobiological parameters for tumor calculations (gEUD and TCP). Values were obtained from AAPM TG‐166 report, Cheung,^54^, ^55^ King,^(56)^ Okunieff,^(45)^ and Levegrun^(57)^ studies

			*Parameters*				
			*a*	$\alpha \;(Gy^{-1})$	α/β (Gy)	$D_{50}(Gy)$	$\gamma_{50}(\%/\%)$
TG‐166	BPhant	PTVRect	‐10	0.1	10	63.3	5
		Rect1	−10	0.1	10	44.2	1.6
	H&N	PTV 70	−10	0.1	10	51.77	2.28
		PTV54	−10	0.1	10	51.77	2.28
		PTV50	−10	0.1	10	51.77	2.28
	Prostate	PTV70.2	−10	0.1	3	46.29	0.95
	Brain	GTV54	−10	0.1	10	22.17	0.70
		PTV50.4	−10	0.1	10	22.17	0.70
Analyzed prostate cases	Cheung and King studies	HR PTV	−10	0.1	1.5	75.5	1.7
		IR PTV	−10	0.1	1.5	67.5	2.2
		LR PTV	−10	0.1	1.5	57.3	1.4
		Bed PTV	−10	0.1	1.5	66.8	2.54
	Okunieff	All staging	−10	0.1	1.5	46.29	0.95
	Levegrun	PTV	−10	0.1	1.5	70.5	2.9

BPhant=benchmark phantom; PTVRect=PTV Rectangle; Rect1=Rectangle 1; H&N=head and neck; HR=high−risk; IR=intermediate−risk; LR=low−risk.

The parameters used for normal tissue calculations (gEUD and NTCP) for TG‐166 test cases and prostate treatments are summarized in Table 2. Values for “a” parameter were extracted from TG‐166 report.^(21)^ The selected “a” values for benchmark phantom case were taken as 12, 1, 4, and 2 for the PTV Rectangle, Rectangle 1, Rectangle 2, and Triangle 1 structures, respectively. These values were selected in order to fit the results from both LKB and Niemierko models (”a” parameter was not reported for the Niemierko model for NTCP calculations in TG‐166 report). Selected α/β values were 3 Gy for all the cases. Values for ${\text{TD}}_{50}$, $\gamma_{50}$, m, and n parameters were shown in the previous report, taken from the study by Burman et al.^(58)^ Additional $\gamma_{50}$ parameters were taken from studies by Stavrev et al.^(59)^ (cord and mandible), Huang et al.^(60)^ (inner ear), and Lee et al.^(61)^ (parotid gland). The $\gamma_{50}$ parameter for the pubic bone was taken as 4, similar to the value for the femoral head. As in the Niemierko model case, m and n parameters from LKB were not reported in the AAPM report; standard values for bone (m=0.12 and n=0.25) were taken in order to perform the calculations.

**Table 2:** Selected radiobiological parameters for normal tissue calculations (gEUD and NTCP). Values were obtained from AAPM TG‐166 report and the studies by Burman et al.,^(58)^ Stavrev et al.,^(59)^ Huang et al.,^(60)^ and Lee et al.(61)

			*Parameters*					
			*a*	α/β (Gy)	$TD_{50}\;(Gy)$	$\gamma_{50}(\%/\%)$	*m*	*n*
TG‐166	BPhant	PTVRect	12	3	80	3	0.12	0.25
		Rect1	1	3	75.1	2.8	0.12	0.25
		Rect2	4	3	55.3	3.1	0.12	0.25
		Triang1	2	3	46	1.8	0.12	0.25
	H&N	Cord	20	3	66.5	2.6	0.175	0.05
		Parotid	1	3	46	2.2	0.18	0.7
		Mandible	10	3	72	3.1	0.1	0.07
	Prostate	Rectum	8	3	80	4	0.15	0.12
		Bladder	8	3	80	4	0.11	0.5
		Fem head	12	3	65	4	0.12	0.25
		PBone	12	3	65	4	0.12	0.25
	Brain	BStem	16	3	65	3	0.14	0.16
		OptCh	16	3	65	3	0.14	0.25
		Eye	16	3	65	2	0.19	0.2
		OptN	16	3	65	3	0.14	0.25
		Inner ear	16	3	65	1.74	0.095	0.01
Prostate cases		Rectum	8.33	3	80	4	0.15	0.12
		Bladder	2	3	80	4	0.11	0.5
		Fem head	4	3	65	4	0.12	0.25

BPhant=benchmark phantom; PTVRect=PTV Rectangle; Rect=Rectangle; Triangl=Triangle1; H&N=head and neck; Fem head=femoral head; PBone=pubic bone; BStem=brain stem; OptCh=optic chiasm; OptN=optic nerve.

### I. Statistical analysis

Results were described as mean ± standard deviation (SD). Data were compared using a paired and two‐tailed Student's *t*‐test. The difference was considered statistically significant for p‐values <0.05. Possible correlation between variables was studied by means of Pearson's correlation coefficient (*r*).

## III. RESULTS

### A. Verifications of biological metrics for both systems with TG‐166 benchmark phantom and test cases

**Figure 2:**
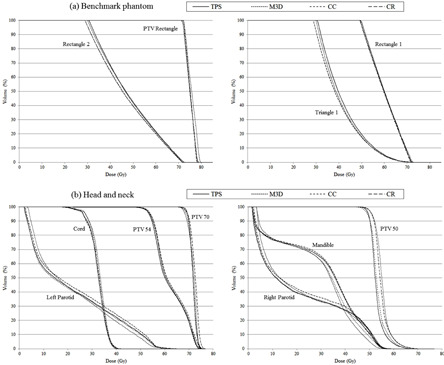
Cumulative dose‐volume histograms for TPS, Mobius3D (M3D), and COMPASS dose calculation (CC) and reconstruction (CR) for two AAPM TG‐166 test cases: (a) benchmark phantom and (b) head and neck case.

**Figure 3:**
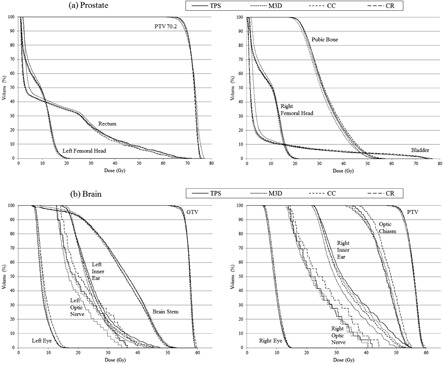
Cumulative dose‐volume histograms for TPS, Mobius3D (M3D), and COMPASS dose calculation (CC) and reconstruction (CR) for two AAPM TG‐166 test cases: (a) prostate and (b) brain cases.

Differences and comparisons between gEUD, TCP, and NTCP calculations from TPS and those obtained from Mobius3D and COMPASS for TG‐166 test cases are shown in Tables 3, 4, and 5, respectively. DVHs from previous cases are plotted in Figs. 2 and 3. Maximum deviations for gEUD were found for left and right inner ear in M3D calculations (8.8% and 9.3%, respectively). CR results were better than M3D and CC results for gEUD discrepancies. The maximum difference for TCP evaluations was found for M3D calculations using the Poisson model (−1.3%). M3D and CR results were better than those from CC. For NTCP, the maximum discrepancy was found in the COMPASS reconstructed dose of Triangle 1 structure from benchmark phantom test (5.3%). There were no statistically significant differences for NTCP comparisons.

**Table 3:** Comparisons of gEUD calculations (target volumes and normal tissues) for TG‐166 test cases between TPS results and those obtained from Mobius3D and COMPASS

		*Differences (%)*		
*TPS Values*	*M3D*	*CC*	*CR*
	*Benchmark Phantom*			
PTV Rectangle (as target)	73.6	−0.8	−0.1	−0.3
PTV Rectangle (as normal tissue)	56.1	0.7	1.0	0.7
Rectangle 1 (as target)	72.1	−1.2	−0.2	−0.1
Rectangle 1 (as normal tissue)	55.7	1.1	−2.2	−2.4
Rectangle 2	50.1	−0.4	1.4	1.5
Triangle 1	40.9	2	2.6	2.7
	*Head & Neck*			
PTV 70	72.2	0.0	0.0	−1.0
PTV 54	59,4	−1.0	−1.1	−1.7
PTV 50	52.9	−4.2	−0.4	−3.3
Cord	35.5	−0.9	−0.7	−1.1
Left Parotid	20.4	0.8	5.2	−2.6
Right Parotid	19.9	−3.4	1.6	−5.0
Mandible	45.3	6.9	2.4	0.5
	*Prostate*			
PTV	69.7	0.3	0.4	0.7
Rectum	42.7	4.1	0.9	4.3
Bladder	43.1	−1.3	−0.2	−2.6
Left Femoral Head	13.9	0.1	3.6	1.0
Right Femoral Head	14.2	−0.5	2.4	1.7
Pubic Bone	39.6	2.8	3.4	−1.9
	*Brain*			
GTV 54	56.3	−0.3	0.2	0.0
PTV 50.4	54.5	0.9	0.1	−0.2
Brain Stem	41.5	0.3	−0.8	1.1
Optic Chiasm	46.5	−0.8	0.0	−2.5
Left Eye	11.0	0.0	1.6	−7.7
Right Eye	11.1	−0.8	1.6	−2.6
Left Optic Nerve	29.5	7.4	1.5	−8.3
Right Optic Nerve	33.8	3.2	2.6	−6.0
Left Inner Ear	35.2	8.8	3.2	5.3
Right Inner Ear	43.5	9.3	4.0	5.5
		M3D vs. CC	M3D vs. CR	CC vs. CR
	p‐values	0.93	<0.05	<0.05

M3D=Mobius3D; CC=COMPASS dose calculation; CR=COMPASS dose reconstruction.

**Table 4:** Comparisons of TCP calculations for TG‐166 test cases between TPS results and those obtained from Mobius3D and COMPASS, using the three described models (Poisson, sigmoidal, and Niemierko)

		*Differences (%)*			
		*TPS Values*	*M3D*	*CC*	*CR*
		*Benchmark Phantom*			
PTV Rectangle	TCP Poisson	82.6	−1.3	−0.1	−0.4
	TCP Sigmoidal	93.3	−0.9	−0.2	−0.3
	TCP Niemierko	95.3	−0.7	−0.1	−0.2
Rectangle 1	TCP Poisson	83.7	0.6	1.2	0.9
	TCP Sigmoidal	84.4	0.4	1.0	0.8
	TCP Niemierko	82.2	0.6	0.9	0.7
		*Head & Neck*			
PTV 70	TCP Poisson	94.4	0.0	0.0	−0.6
	TCP Sigmoidal	95.0	0.0	0.0	−0.5
	TCP Niemierko	95.4	0.0	0.0	−0.4
		*Prostate*			
PTV 70.2	TCP Poisson	98.7	0.1	0.1	0.1
	TCP Sigmoidal	84.6	0.2	0.3	0.7
	TCP Niemierko	82.6	0.2	0.2	0.4
		*Brain*			
GTV 54	TCP Poisson	98.9	0.0	0.0	0.0
	TCP Sigmoidal	97.0	−0.1	0.1	0.0
	TCP Niemierko	93.2	0.0	0.0	0.0
		p‐values	M3Dvs.CC<0.05	M3Dvs.CR<0.05	CC vs.CR<0.05

M3D=Mobius3D; CC=COMPASS dose calculation; CR=COMPASS dose reconstruction.

**Table 5:** Comparisons of NTCP calculations for TG‐166 test cases between TPS results and those obtained from Mobius3D and COMPASS, using the two described models (Lyman‐Kutcher‐Burman and Niemierko)

	*NTCP LKB*				*NTCP Niemierko*			
		*Differences (%)*			*Differences (%)*			
	*TPS Values*	*M3D*	*CC*	*CR*	*TPS values*	*M3D*	*CC*	*CR*
*Benchmark Phantom*							
PTV R	19.5	−3.2	−1.1	−0.3	22.4	−2.7	−0.4	−0.2
Rect1	3.1	0.2	−0.1	−0.2	3.4	0.4	−0.9	−1.0
Rect2	21.6	−1.0	2.9	3.1	22.6	−1.0	2.8	3.0
Triang1	29.3	4.1	5.0	5.3	30.1	2.9	3.7	3.8
*Head & Neck*							
Cord	$38.7\cdot 10^{-2}$	$-3.2\cdot 10^{-2}$	$-2.4\cdot 10^{-2}$	$-4.3\cdot 10^{-2}$	$14.6\cdot 10^{-2}$	$-1.4\cdot 10^{-2}$	$-1.0\cdot 10^{-2}$	$-1.9\cdot 10^{-2}$
L Parot	$38.0\cdot 10^{-2}$	$8.1\cdot 10^{-2}$	$9.7\cdot 10^{-2}$	$-8.9\cdot 10^{-2}$	$7.9\cdot 10^{-2}$	$0.5\cdot 10^{-2}$	$2.8\cdot 10^{-2}$	$-2.0\cdot 10^{-2}$
R Parot	$38.3\cdot 10^{-2}$	$-4.0\cdot 10^{-2}$	$3.1\cdot 10^{-2}$	$-8.6\cdot 10^{-2}$	$6.3\cdot 10^{-2}$	$-2.2\cdot 10^{-2}$	$0.8\cdot 10^{-2}$	$-3.6\cdot 10^{-2}$
Mandbl	$3.2\cdot 10^{-2}$	$2.5\cdot 10^{-2}$	$1.3\cdot 10^{-2}$	$0.5\cdot 10^{-2}$	$32.0\cdot 10^{-2}$	$18\cdot 10^{-2}$	$8.2\cdot 10^{-2}$	$2.1\cdot 10^{-2}$
	*Prostate*							
Rectum	$11.1\cdot 10^{-2}$	$4.2\cdot 10^{-2}$	$1.1\cdot 10^{-2}$	$4.4\cdot 10^{-2}$	$4.4\cdot 10^{-3}$	$2.1\cdot 10^{-3}$	$0.6\cdot 10^{-3}$	$2.2\cdot 10^{-3}$
Bladder	$3.1\cdot 10^{-12}$	$1.2\cdot 10^{-12}$	$0.2\cdot 10^{-12}$	$3.7\cdot 10^{-12}$	$5.0\cdot 10^{-3}$	$-1.2\cdot 10^{-3}$	$-0.2\cdot 10^{-3}$	$-2.6\cdot 10^{-3}$
L FemH	$24.5\cdot 10^{-11}$	$-5.2\cdot 10^{-11}$	$6.6\cdot 10^{-11}$	$0.9\cdot 10^{-11}$	$18.6\cdot 10^{-10}$	$0.2\cdot 10^{-10}$	$8.1\cdot 10^{-10}$	$2.8\cdot 10^{-10}$
R FemH	$4.0\cdot 10^{-10}$	$-1.2\cdot 10^{-10}$	$0.8\cdot 10^{-10}$	$0.1\cdot 10^{-10}$	$26.9\cdot 10^{-10}$	$-2.3\cdot 10^{-10}$	$8.5\cdot 10^{-10}$	$6.4\cdot 10^{-10}$
PBone	$3.4\cdot 10^{-3}$	$0.8\cdot 10^{-3}$	$1.6\cdot 10^{-3}$	$-1.2\cdot 10^{-3}$	$3.5\cdot 10^{-2}$	$1.3\cdot 10^{-2}$	$1.5\cdot 10^{-2}$	$-1.3\cdot 10^{-2}$
	*Brain*							
BStem	$14.8\cdot 10^{-2}$	$1.2\cdot 10^{-2}$	$-2.2\cdot 10^{-2}$	$1.9\cdot 10^{-2}$	$4.6\cdot 10^{-2}$	$1.5\cdot 10^{-2}$	$-4.7\cdot 10^{-2}$	$5.6\cdot 10^{-2}$
OptCh	$10.3\cdot 10^{-1}$	$-1.1\cdot 10^{-1}$	$-0.3\cdot 10^{-1}$	$-4.9\cdot 10^{-1}$	$17.6\cdot 10^{-1}$	$-1.7\cdot 10^{-1}$	$0.0\cdot 10^{-1}$	$-6.1\cdot 10^{-1}$
L Eye	$25.7\cdot 10^{-5}$	$-2.7\cdot 10^{-5}$	$0.6\cdot 10^{-5}$	$-10\cdot 10^{-5}$	$6.8\cdot 10^{-5}$	$0.0\cdot 10^{-5}$	$0.8\cdot 10^{-5}$	$-6.1\cdot 10^{-5}$
R Eye	$36.0\cdot 10^{-5}$	$-3.0\cdot 10^{-5}$	$1.0\cdot 10^{-5}$	$-5.6\cdot 10^{-5}$	$7.2\cdot 10^{-5}$	$-0.5\cdot 10^{-5}$	$0.9\cdot 10^{-5}$	$-1.7\cdot 10^{-5}$
L OptN	$2.8\cdot 10^{-4}$	$2.0\cdot 10^{-4}$	$0.8\cdot 10^{-4}$	$-5.7\cdot 10^{-4}$	$7.7\cdot 10^{-3}$	$4.4\cdot 10^{-3}$	$1.3\cdot 10^{-3}$	$-14\cdot 10^{-3}$
R OptN	$1.2\cdot 10^{-3}$	$0.2\cdot 10^{-3}$	$0.3\cdot 10^{-3}$	$-2.6\cdot 10^{-3}$	$3.9\cdot 10^{-2}$	$1.2\cdot 10^{-2}$	$1.1\cdot 10^{-2}$	$-4.3\cdot 10^{-2}$
L InnE	$1.8\cdot 10^{-2}$	$1.5\cdot 10^{-2}$	$1.1\cdot 10^{-2}$	$1.3\cdot 10^{-2}$	$13.8\cdot 10^{-1}$	$6.1\cdot 10^{-1}$	$2.7\cdot 10^{-1}$	$4.2\cdot 10^{-1}$
R InnE	$8.6\cdot 10^{-1}$	$6.4\cdot 10^{-1}$	$3.9\cdot 10^{-1}$	$5.5\cdot 10^{-1}$	5.8	2.6	1.3	1.7
			M3D vs. CC		M3D vs. CR		CC vs. CR	
		p‐values	0.11		0.12		0.50	

M3D=Mobius3D; CC=COMPASS dose calculation; CR=COMPASS dose reconstruction; PTV R=PTV Rectangle; Rect=Rectangle; Triangl=Triangle 1; Parot=parotid glands; Mandbl=mandible; FemH=femoral heads; Pbone=pubic bone; Bstem=brain stem; OptCh=optic chiasm; OptN=optic nerve; InnE=inner ear.

### B. Evaluation of prostate treatments with classical (gamma) and DVH‐based dose‐volume metrics

Mean global gamma passing rates for all structures are shown in Table 6. Considering mean values for all structures, M3D passing rates were worse than those from COMPASS in all cases, with the exception of values for 3%/3 mm criterion in low‐risk treatments (mean passing rates for all structures were 99.8% for both M3D and CR results).

TPS mean values of the dosimetric parameters analyzed for each prostate case are shown in Table 7. Mean differences and comparisons between previous values and results for the same parameters determined by M3D, CC, and CR are also presented in this table. Maximum discrepancies for PTVs were found in COMPASS reconstructed low‐risk and prostate bed patients (2.4%±0.8%). The largest differences observed for rectum were found for COMPASS reconstructed mean dose in bed patients (6.2%±1.3%). For bladder, the maximum differences were also located in mean dose Mobius3D calculated values for bed treatments (−3.3%±1.6%). The worst mean differences for femoral heads were found for CR results for the right head in low‐risk patients (3.1%±1.3%). CR results were worse (p<0.05) than M3D and CC results for a huge number of cases. Statistically significant differences for comparisons between M3D and CC were also observed. Deviations were better for M3D than CC in some cases, and vice versa. For all the parameters, mean values were 0.5%±1.9%, 0.4%±1.0%, and 1.1%±2.6% for M3D, CC, and CR, respectively. Considering all parameters, M3D results were better than CR results, and CC values were better than those obtained by M3D and CR.

**Table 6:** Mean gamma passing rates (2%/2mm and 3%/3mm, global normalization to maximum with a low‐dose threshold at 10% of global maximum) for 100 analyzed prostate treatments, 25 from each group (high‐, intermediate‐, low‐risk, and prostate bed patients)

		*Mean Global Gamma Passing Rates (%)*					
		Gamma 2%/2mm			Gamma 3%/3mm		
		*M3D*	*CC*	*CR*	*M3D*	*CC*	*CR*
HR	Prostate PTV	85.4±8.5	93.7±3.4	86±13	98.9±2.9	99.5±0.7	98.3±4.2
	SV PTV	95.3±4.7	99.4±1.0	96.2±4.2	99.2±1.9	100.0±0.0	99.8±1.0
	LN PTV	55±13	86.8±4.1	90.9±6.6	88.9±7.8	97.3±1.9	99.4±0.9
	Rectum	91.2±5.5	99.3±1.1	96.3±4.0	99.2±1.2	99.9±0.3	99.9±0.2
	Bladder	86.7±7.0	98.5±1.8	97.4±5.7	98.1±2.2	99.9±0.2	99.8±0.8
	L Fem Head	98.5±1.7	98.2±1.6	97.2±2.4	100.0±0.1	100.0±0.0	100.0±0.1
	R Fem Head	97.8±2.5	98.2±1.8	94.7±3.7	100.0±0.0	100.0±0.0	99.9±0.2
	Whole Vol	93.5±1.6	97.8±0.8	98.1±1.2	99.3±0.4	99.7±0.2	99.9±0.2
IR	Prostate PTV	92.9±6.9	99.3±1.0	96.3±2.8	99.1±1.5	99.9±0.1	99.7±0.8
	SV PTV	91.8±7.5	99.8±0.4	97.2±3.0	99.2±1.9	100.0±0.0	99.9±0.2
	Rectum	90.1±5.0	99.9±0.1	93.0±4.1	99.7±0.4	100.0±0.0	99.8±0.6
	Bladder	92.2±5.7	99.9±0.1	99.3±1.0	100.0±0.1	100.0±0.0	100.0±0.0
	L Fem Head	97.7±4.4	99.7±0.8	95.8±3.8	99.9±0.2	100.0±0.0	99.8±0.6
	R Fem Head	97.4±3.9	99.6±1.1	92.9±5.8	99.5±2.1	100.0±0.0	99.5±0.9
	Whole Vol	98.7±0.3	99.8±0.9	99.1±1.7	99.9±0.1	99.8±0.9	99.6±1.7
LR	Prostate PTV	92.6±5.9	99.5±0.4	96.0±2.8	99.5±1.6	99.9±0.1	99.8±0.4
	Rectum	92.6±3.8	99.8±0.6	93.7±3.8	99.9±0.4	100.0±0.0	99.9±0.2
	Bladder	86.4±8.7	99.1±1.0	99.4±0.7	100.0±0.0	100.0±0.1	99.9±0.3
	L Fem Head	99.0±1.1	99.8±0.5	98.2±2.3	100.0±0.1	100.0±0.0	99.9±0.3
	R Fem Head	98.5±1.9	99.6±0.7	96.0±4.1	99.9±0.2	100.0±0.1	99.7±1.2
	Whole Vol	98.7±0.4	99.9±0.0	99.5±0.6	100.0±0.1	100.0±0.0	99.9±0.2
Bed	Bed PTV	87.0±7.8	98.0±0.9	96.2±2.3	98.0±2.1	99.9±0.1	99.8±0.3
	Rectum	86.9±4.6	99.6±0.9	91.0±5.6	99.2±1.4	100.0±0.0	99.6±0.7
	Bladder	83.5±7.1	99.6±0.3	99.3±1.0	99.8±0.4	100.0±0.0	100.0±0.1
	L Fem Head	98.4±1.6	99.7±0.6	96.3±3.1	99.9±0.3	100.0±0.0	99.9±0.3
	R Fem Head	98.6±1.3	99.7±0.3	93.7±3.6	100.0±0.1	100.0±0.0	99.6±0.7
	Whole Vol	97.7±0.6	99.9±0.0	99.3±0.3	99.9±0.1	100.0±0.0	100.0±0.1

M3D=Mobius3D; CC=COMPASS dose calculation; CR=COMPASS dose reconstruction; HR=high−risk; IR=intermediate−risk; LR=low−risk; SV=seminal vesicles; LN=pelvic lymph nodes; Fem Head=femoral heads; Whole Vol=whole volume.

**Table 7:** Differences and comparisons for dosimetric parameters (target volumes and normal tissues) for 100 analyzed prostate treatments, 25 from each group (high‐, intermediate‐, low‐risk, and prostate bed patients)

			Mean Differences (%)			p‐values		
		*TPS Mean Values*	*M3D*	*CC*	*CR*	*M3D vs. CC*	*M3D vs. CR*	*CC vs. CR*
*High‐risk*								
Prost PTV	$D_{98}$	78.3±0.6	1.2±1.1	0.2±0.4	0.8±0.8	<0.05	0.16	<0.05
	$D_{2}$	85.2±0.5	−0.9±0.8	−1.3±0.6	−1.3±0.6	<0.05	<0.05	0.70
	$D_{50}$	82.6±0.4	−0.6±0.6	−0.6±0.3	−1.1±0.6	0.92	<0.05	<0.05
	$D_{m}$	82.4±0.4	−0.4±0.6	−0.6±0.3	1.5±12.2	0.13	0.44	0.40
SV PTV	$D_{98}$	63.1±1.9	0.1±1.6	−0.2±0.6	1.9±1.4	0.28	<0.05	<0.05
	$D_{2}$	84.0±0.7	−0.1±0.8	−0.3±0.6	−0.7±0.9	0.16	<0.05	<0.05
	$D_{50}$	75.2±3.9	0.4±1.1	0.0±0.5	0.8±1.2	0.06	0.18	<0.05
	$D_{m}$	74.8±2.4	0.1±0.9	−0.1±0.4	0.4±0.9	0.39	0.17	<0.05
LN PTV	$D_{98}$	54.6±0.5	−1.1±1.2	2.3±0.9	−0.1±0.8	<0.05	<0.05	<0.05
	$D_{2}$	67.5±5.6	−0.7±0.9	−1±0.4	−0.9±0.8	0.06	0.30	0.61
	$D_{50}$	58.7±0.4	−2.7±0.7	−0.5±0.3	−1.2±0.5	<0.05	<0.05	<0.05
	$D_{m}$	59.2±1.2	−2.3±0.7	−0.2±0.2	−1.0±0.5	<0.05	<0.05	<0.05
Rectum	$V_{50}$	25.5±5.9	1.3±1.9	0.6±0.8	3.2±1.8	<0.05	<0.05	<0.05
	$V_{60}$	14.5±5.2	1.9±1.3	0.5±0.4	2.6±1.1	<0.05	<0.05	<0.05
	$V_{65}$	10.9±4.3	1.8±1.2	0.3±0.3	2.2±0.9	<0.05	<0.05	<0.05
	$V_{70}$	7.9±3.6	1.8±1.2	0.3±0.3	2.0±0.9	<0.05	0.24	<0.05
	$V_{75}$	4.4±2.8	1.5±1.4	0.1±0.4	1.5±0.9	<0.05	0.71	<0.05
	$D_{M}$	41.2±2.9	0.6±2.1	1.1±0.9	3.9±1.8	0.16	<0.05	<0.05
Bladder	$V_{65}$	17.9±6.4	−0.1±0.9	0.3±0.3	−1.0±0.7	<0.05	<0.05	<0.05
	$V_{70}$	13.3±5.1	0.1±0.7	0.2±0.3	−1.0±0.7	0.27	<0.05	<0.05
	$V_{75}$	8.3±3.6	0.1±0.8	0.2±0.4	−1.1±0.8	0.69	<0.05	<0.05
	$V_{80}$	1.5±1.3	−0.7±1.1	−0.3±0.5	−1.8±1.8	<0.05	<0.05	<0.05
	$D_{M}$	51±3.2	−1.6±0.9	0.4±0.4	−1.2±0.8	<0.05	0.13	<0.05
L FemH	$D_{\text{max}}$	46.2±1.5	2.6±2.1	2.9±0.9	2.2±1.2	0.47	0.40	<0.05
R FemH	$D_{\text{max}}$	46.9±1.2	2.3±1.7	2.7±1.0	2.8±1.1	0.19	0.25	0.75
*Intermediate‐risk*								
Prost PTV	$D_{98}$	78.5±0.9	1.9±1.3	0.3±0.4	2.3±0.7	<0.05	0.10	<0.05
	$D_{2}$	85.0±0.4	0.0±0.6	−0.3±0.3	0.0±0.4	<0.05	0.59	<0.05
	$D_{50}$	82.8±0.5	0.0±0.6	0.0±0.3	0.1±0.4	0.66	0.66	0.26
	$D_{m}$	82.5±0.5	0.4±0.9	0.0±0.3	0.2±0.4	<0.05	0.46	<0.05
SV PTV	$D_{98}$	62.4±1.3	0.0±1.7	−0.3±0.4	1.8±1.2	0.33	<0.05	<0.05
	$D_{2}$	83.7±0.7	−0.1±0.8	−0.2±0.3	0.1±0.7	0.69	0.16	0.06
	$D_{50}$	73.7±3.6	−0.2±1.1	−0.1±0.4	0.9±1.1	0.42	<0.05	<0.05
	$D_{m}$	73.9±2.2	−0.3±0.9	−0.2±0.4	0.7±0.8	0.27	<0.05	<0.05
Rectum	$V_{50}$	24.6±6.4	1.5±0.9	0.6±0.4	3.8±0.9	<0.05	<0.05	<0.05
	$V_{60}$	15.4±4.8	1.9±0.9	0.4±0.4	3.3±0.9	<0.05	<0.05	<0.05
	$V_{65}$	11.3±4	1.9±0.8	0.4±0.3	2.8±0.9	<0.05	<0.05	<0.05
	$V_{70}$	8.1±3.3	1.9±0.9	0.3±0.3	2.6±1.0	<0.05	<0.05	<0.05
	$V_{75}$	4.4±2.4	1.6±1.0	0.2±0.4	2.2±1.0	<0.05	<0.05	<0.05
	$D_{M}$	36.4±3.6	−0.3±1.4	1.1±0.8	5.5±1.5	<0.05	<0.05	<0.05
Bladder	$V_{65}$	13.1±5.8	0.3±0.7	0.3±0.2	−1.2±0.6	0.71	<0.05	<0.05
	$V_{70}$	10.2±4.6	0.4±0.7	0.2±0.2	−1.1±0.6	0.08	<0.05	<0.05
	$V_{75}$	6.8±3.1	0.3±0.7	0.1±0.3	−1.3±0.7	0.08	<0.05	<0.05
	$V_{80}$	0.8±0.7	−0.8±0.8	−0.3±0.4	−1.1±0.7	<0.05	<0.05	<0.05
	$D_{M}$	30.1±8.2	−2.7±2.1	2.1±0.8	−2.0±1.2	<0.05	0.23	<0.05
L FemH	$D_{\text{max}}$	36.2±4.8	2.0±1.7	2.2±0.8	3.1±1.6	0.50	<0.05	<0.05
R FemH	$D_{\text{max}}$	36.3±4.9	1.3±1.6	1.9±0.7	2.9±1.3	<0.05	<0.05	<0.05
*Low‐risk*								
Prost PTV	$D_{98}$	78.6±0.9	2.1±1.5	0.2±0.4	2.4±0.8	<0.05	0.33	<0.05
	$D_{2}$	84.9±0.6	−0.2±0.7	−0.3±0.2	0.1±0.5	0.18	0.06	<0.05
	$D_{50}$	82.8±0.6	−0.2±0.5	−0.2±0.2	0.1±0.4	0.53	<0.05	<0.05
	$D_{m}$	82.5±0.6	0.1±0.6	−0.2±0.2	0.3±0.4	<0.05	0.17	<0.05
Rectum	$V_{50}$	22.4±6.4	1.2±0.8	0.5±0.3	3.1±0.7	<0.05	<0.05	<0.05
	$V_{60}$	15.8±5.1	1.7±0.8	0.4±0.2	2.8±0.7	<0.05	<0.05	<0.05
	$V_{65}$	12.9±4.6	1.9±0.9	0.4±0.3	2.9±0.9	<0.05	<0.05	<0.05
	$V_{70}$	9.9±3.9	2.0±0.9	0.4±0.3	2.7±0.9	<0.05	<0.05	<0.05
	$V_{75}$	6.2±3.0	1.9±1.1	0.2±0.4	2.6±1	<0.05	<0.05	<0.05
	$D_{M}$	32.6±5.4	−0.5±1.8	1.4±0.6	6.0±1.1	<0.05	<0.05	<0.05
Bladder	$V_{65}$	14.6±6.9	0.8±0.7	0.4±0.2	−0.9±0.7	<0.05	<0.05	<0.05
	$V_{70}$	11.8±5.7	0.8±0.8	0.3±0.2	−0.9±0.6	<0.05	<0.05	<0.05
	$V_{75}$	8.1±4.2	0.6±0.8	0.2±0.3	−0.9±0.6	<0.05	<0.05	<0.05
	$V_{80}$	1.5±1.2	−0.8±0.9	−0.4±0.4	−0.7±0.9	<0.05	0.56	0.18
	$D_{M}$	28.7±9.8	−3.7±3.1	2.5±0.7	−1.5±1.5	<0.05	<0.05	<0.05
L FemH	$D_{\text{max}}$	35.2±4.3	1.1±1.6	1.7±0.5	2.2±2.6	0.07	0.07	0.33
R FemH	$D_{\text{max}}$	34.7±4.1	0.5±1.4	1.7±0.7	3.1±1.3	<0.05	<0.05	<0.05
Prostate Bed								
Bed PTV		71.0±0.9	2.1±1.5	0.0±0.5	2.4±0.8	<0.05	0.13	<0.05
	$D_{2}$	78.1±0.4	0.3±0.7	−0.4±0.4	0.2±0.5	<0.05	0.47	<0.05
	$D_{50}$	75.4±0.4	−0.1±0.8	−0.2±0.3	0.0±0.4	0.17	0.53	<0.05
	$D_{m}$	75.2±0.4	0.2±0.8	−0.2±0.3	0.2±0.4	<0.05	0.91	<0.05
Rectum	$V_{50}$	32.0±7.0	2.3±1.2	0.7±0.3	4.3±1.0	<0.05	<0.05	<0.05
	$V_{60}$	22.9±5.6	3.2±1.4	0.6±0.5	4.3±1.2	<0.05	<0.05	<0.05
	$V_{65}$	17.8±4.9	3.4±1.6	0.5±0.6	4.2±1.4	<0.05	<0.05	<0.05
	$V_{70}$	11.4±3.9	3.3±2.0	0.2±0.9	3.7±1.6	<0.05	0.08	<0.05
	$V_{75}$	2.4±1.9	1.4±1.4	0.0±1.0	1.4±1.1	<0.05	0.96	<0.05
	$D_{M}$	37.5±5	0.8±1.7	1.3±0.8	6.2±1.3	0.09	<0.05	<0.05
Bladder	$V_{65}$	25.6±7.2	−0.1±0.4	0.1±0.1	−1.0±0.7	<0.05	<0.05	<0.05
	$V_{70}$	22.1±6.4	−0.2±0.5	0.0±0.2	−1.2±0.7	<0.05	<0.05	<0.05
	$V_{75}$	9.9±4.6	−2.8±2.8	−0.7±1.8	−2.1±2.4	<0.05	0.11	<0.05
	$V_{80}$	0.0±0.0	−0.2±0.8	−0.1±0.5	0.0±0.0	0.78	0.32	0.31
	$D_{M}$	35.4±8.0	−3.3±1.6	1.2±0.6	−0.6±1.3	<0.05	<0.05	<0.05
L FemH	$D_{\text{max}}$	41.5±4.1	2.4±1.7	2.2±1.0	2.6±1.2	0.67	0.37	0.05
R FemH	$D_{\text{max}}$	41.2±3.4	2.7±1.7	2.4±0.6	2.8±1.4	0.27	0.66	0.09

M3D=Mobius3D; CC=COMPASS dose calculation; CR=COMPASS dose reconstruction; Prost=prostate; SV=seminal vesicles; LN=pelvic lymph nodes; FemH=femoral heads.

### C. Biological metrics applied prostate treatments

Discrepancies in gEUD calculations between TPS, dose calculation and reconstruction for all prostate cases are shown in Table 8, taking previous reported data from different studies. Maximum mean differences were found for rectum COMPASS reconstructed gEUD for intermediate‐ and low‐risk patients (4.7%±1.3%). Prostate gland absolute mean differences were lower than 1.3% in all cases; the maximum mean discrepancy for bladder was found in intermediate‐risk COMPASS reconstructed cases (−2.5%±1.3%); the worst femoral head deviation was also found in the intermediate‐risk COMPASS reconstructed (right femoral head) cases (4.2%±1.4%). CC results were better than both CR and M3D results for prostate gland values in low‐risk treatments, for all rectum values, and for bladder values in high‐risk and prostate bed treatments. In addition, CC results were better than CR results for prostate gland values in high‐risk treatments, for bladder values in low‐ and intermediate‐risk treatments, and for all femoral head values (excepting left head values for high‐risk treatments). M3D results were better than both CR and M3D results for bladder values in low‐ and intermediate‐risk treatments and for

**Table 8:** Calculations and comparisons of gEUD values for 100 analyzed prostate treatments, 25 from each group (high‐, intermediate‐, low‐risk, and prostate bed patients)

			*Prostate*	*Rectum*	*Bladder*	*R Fem Head*	*L Fem Head*
HR	gEUD mean value (Gy)		83.1±0.5	57.2±2.9	52.1±3.0	27.5±2.3	26.7±2.4
	Mean	M3D	−1.0±0.7	2.8±2.0	−1.3±0.8	1.9±1.6	2.0±1.7
	difference (%)	CC	−0.9±0.4	0.1±0.7	0.4±0.6	3.2±0.7	3.6±0.7
		CR	−1.3±0.7	3.2±1.1	−1.3±1.0	3.6±1.0	3.1±1.3
	p‐values	M3D vs. CC	0.4	<0.05	<0.05	<0.05	<0.05
		M3D vs. CR	0.12	0.23	0.97	<0.05	<0.05
		CC vs. CR	<0.05	<0.05	<0.05	<0.05	0.07
IR	gEUD mean value (Gy)		83.3±0.6	57.3±2.8	37.2±6.8	21.8±3.2	21.2±3.0
	Mean	M3D	−0.1±0.6	3.1±1.6	−0.5±1.4	1.6±1.7	1.3±1.1
	difference (%)	CC	−0.1±0.3	0.3±0.7	1.3±0.7	2.4±0.7	2.3±0.8
		CR	0.1±0.3	4.7±1.3	−2.5±1.3	4.2±1.4	3.6±1.1
	p‐values	M3D vs. CC	0.53	<0.05	<0.05	<0.05	<0.05
		M3D vs. CR	0.12	<0.05	<0.05	<0.05	<0.05
		CC vs. CR	0.13	<0.05	<0.05	<0.05	<0.05
LR	gEUD mean value (Gy)		83.2±0.7	58.6±2.4	37.0±8.0	20.8±2.8	20.7±2.9
	Mean	M3D	−0.3±0.7	3.4±2.0	−0.3±1.8	0.7±1.2	0.7±1.3
	difference (%)	CC	−0.1±0.3	0.5±0.7	1.4±0.8	2.0±0.6	2.0±0.5
		CR	0.2±0.5	4.7±1.3	−1.9±1.2	3.7±0.9	2.9±1.3
	p‐values	M3D vs. CC	<0.05	<0.05	<0.05	<0.05	<0.05
		M3D vs. CR	<0.05	<0.05	<0.05	<0.05	<0.05
		CC vs. CR	<0.05	<0.05	<0.05	<0.05	<0.05
Bed	gEUD mean value (Gy)		75.7±0.5	58.8±2.1	44.1±5.9	23.2±2.6	23.5±3.1
Mean	M3D	−0.2±0.9	3.1±1.7	−1.3±1.0	1.2±1.2	1.2±1.2
	difference (%)	CC	−0.3±0.5	0.3±0.9	0.4±0.9	2.3±0.6	2.2±0.7
		CR	0.0±0.5	3.7±1.2	−1.2±1.2	3.9±1.3	3.7±1.1
	p‐values	M3D vs. CC	0.71	<0.05	<0.05	<0.05	<0.05
		M3D vs. CR	0.08	<0.05	0.75	<0.05	<0.05
		CC vs. CR	<0.05	<0.05	<0.05	<0.05	<0.05

M3D=Mobius3D; CC=COMPASS dose calculation; CR=COMPASS dose reconstruction; HR=high−risk; IR=intermediate−risk; LR=low−risk.

all femoral head values. In addition, M3D results were better than CR ones for rectum values in intermediate‐risk treatments. Finally, CR results were better than M3D results for prostate gland values in low‐risk treatments and also better than CC results for prostate gland values in prostate bed treatments. The remaining differences were not statistically significant.

TCP comparisons between the TPS, M3D, and COMPASS for all the previously described models are shown in Table 9. The maximum mean difference was found for high‐risk COMPASS reconstructed values using the Poisson model and taking the values for radiobiological parameters from the study by Cheung et al.^(54)^ (−2.4%±1.1%). CC results were better than CR results for all high‐risk treatments (except those obtained using the sigmoidal model and taking the values from the study by Okunieff et al.^(45)^ and prostate bed values. CC and CR results were better than M3D ones for all low‐risk values. In addition, CC results were better than M3D results using the Niemierko model with any set of parameters and using the sigmoidal model with the parameters by Levegrun et al.^(57)^ The remaining differences for TCP comparisons were not statistically significant.

NTCP comparisons between the TPS and the two systems are shown in Table 10. The worst mean difference was found for COMPASS reconstructed rectum values in low‐risk treatments (1.5%±0.5%). Discrepancies for bladder and femoral heads were better than 0.1% and 0.01%, respectively, in all cases. CC results were better than both CR and M3D results for rectum values in all cases, for bladder values in high‐risk and prostate bed treatments and also for intermediate‐risk treatments using the Niemierko model. In addition, CC results were better than CR ones for left femoral head values and better than M3D results for bladder values in intermediate‐risk treatments using the Niemierko model. M3D results were better than both CC and CR results in the same previous case using both LKB and Niemierko models. M3D results were better than CC ones for left femoral head values in high‐risk (Niemierko model) and prostate bed treatments (LKB and Niermierko models). Finally, M3D results were better than CR results for rectum values in intermediate‐ and low‐risk, as well as prostate bed treatments, for right femoral head values using both models and for left femoral head values using the Niemierko model in prostate bed treatments. There was no statistically significant difference for other NTCP comparisons.

**Table 9:** Calculations and comparisons of TCP values for 100 analyzed prostate treatments, 25 from each group (high‐, intermediate‐, low‐risk, and prostate bed patients), using the three models and set of parameters described in the text

		*Mean Differences (%)*			*p‐values*		
*Parameters*	*Model*	*M3D*	*CC*	*CR*	*M3D vs. CC*	*M3D vs. CR*	*CC vs. CR*
		*High‐risk Prostate*					
Cheung	Poisson	−2.0±1.2	−1.6±0.8	−2.4±1.1	0.13	0.19	<0.05
	Sigmoidal	−1.5±1.2	−1.5±0.8	−1.9±1.2	0.90	0.12	<0.05
	Niemierko	−1.5±1.0	−1.4±0.6	−1.9±1.0	0.41	0.12	<0.05
Okunieff	Poisson	$(-2.4\pm 1.5)\cdot 10^{-3}$	$(-1.9\pm 1.0)\cdot 10^{-3}$	$(-2.8\pm 1.4)\cdot 10^{-3}$	0.15	0.19	<0.05
	Sigmoidal	−0.4±0.3	−0.3±0.2	−0.5±0.3	0.99	0.12	0.06
	Niemierko	−0.3±0.2	−0.3±0.1	−0.4±0.2	0.42	0.12	<0.05
Levegrun	Poisson	−0.7±0.4	−0.5±0.3	−0.8±0.4	0.14	0.19	<0.05
	Sigmoidal	−1.3±0.9	−1.2±0.5	−1.6±0.8	0.49	0.12	<0.05
	Niemierko	−1.3±0.9	−1.1±0.5	−1.6±0.8	0.47	0.12	<0.05
		*Intermediate‐risk Prostate*					
Cheung	Poisson	$(-0.4\pm 2.1)\cdot 10^{-1}$	$(0.6\pm 13)\cdot 10^{-2}$	$(0.5\pm 1.4)\cdot 10^{-1}$	0.21	0.06	0.14
	Sigmoidal	$(-1.3\pm 7.4)\cdot 10^{-1}$	$(-0.7\pm 3.8)\cdot 10^{-1}$	$(0.7\pm 3.8)\cdot 10^{-1}$	0.63	0.19	0.17
	Niemierko	$(-1.3\pm 6.7)\cdot 10^{-1}$	$(-0.6\pm 3.6)\cdot 10^{-1}$	$(0.8\pm 3.6)\cdot 10^{-1}$	0.54	0.13	0.12
Okunieff	Poisson	$(-0.3\pm 1.5)\cdot 10^{-3}$	$(0.5\pm 9.4)\cdot 10^{-4}$	$(0.4\pm 1.0)\cdot 10^{-3}$	0.21	0.06	0.14
	Sigmoidal	$(-0.4\pm 3.0)\cdot 10^{-1}$	$(-0.3\pm 1.8)\cdot 10^{-1}$	$(0.1\pm 1.6)\cdot 10^{-1}$	0.77	0.38	0.38
	Niemierko	$(-0.4\pm 2.2)\cdot 10^{-1}$	$(-0.2\pm 1.1)\cdot 10^{-1}$	$(0.3\pm 1.2)\cdot 10^{-1}$	0.53	0.13	0.12
Levegrun	Poisson	$(-0.8\pm 4.1)\cdot 10^{-1}$	$(0.1\pm 2.6)\cdot 10^{-1}$	$(1.0\pm 2.8)\cdot 10^{-1}$	0.21	0.05	0.14
	Sigmoidal	$(-1.7\pm 8.4)\cdot 10^{-1}$	$(-0.8\pm 4.2)\cdot 10^{-1}$	(1.0±4.5)⋅10^‐1^	0.56	0.14	0.12
	Niemierko	$(-1.6\pm 8.4)\cdot 10^{-1}$	$(-0.7\pm 4.1)\cdot 10^{-1}$	$(1.0\pm 4.5)\cdot 10^{-1}$	0.55	0.14	0.12
		*Low‐risk Prostate*					
Cheung	Poisson	$(-0.9\pm 2.0)\cdot 10^{-2}$	$(-4.0\pm 8.1)\cdot 10^{-3}$	$(0.4\pm 1.7)\cdot 10^{-2}$	0.13	<0.05	<0.05
	Sigmoidal	$(-1.7\pm 4.8)\cdot 10^{-1}$	$(-0.6\pm 2.9)\cdot 10^{-1}$	$(1.5\pm 3.8)\cdot 10^{-1}$	0.09	<0.05	<0.05
	Niemierko	$(-1.7\pm 3.6)\cdot 10^{-1}$	$(-0.7\pm 1.9)\cdot 10^{-1}$	$(1.0\pm 3.0)\cdot 10^{-1}$	<0.05	<0.05	<0.05
Okunieff	Poisson	$(-0.7\pm 1.6)\cdot 10^{-3}$	$(-3.1\pm 6.2)\cdot 10^{-4}$	$(0.3\pm 1.3)\cdot 10^{-3}$	0.13	<0.05	<0.05
	Sigmoidal	$(-1.0\pm 3.2)\cdot 10^{-1}$	$(-0.3\pm 2.1)\cdot 10^{-1}$	$(1.0\pm 2.5)\cdot 10^{-1}$	0.12	<0.05	<0.05
	Niemierko	$(-1.1\pm 2.2)\cdot 10^{-1}$	$(-0.5\pm 1.1)\cdot 10^{-1}$	$(0.6\pm 1.8)\cdot 10^{-1}$	<0.05	<0.05	<0.05
Levegrun	Poisson	$(-2.0\pm 4.3)\cdot 10^{-1}$	$(-0.9\pm 1.7)\cdot 10^{-1}$	$(0.9\pm 3.7)\cdot 10^{-1}$	0.12	<0.05	<0.05
	Sigmoidal	$(-4.0\pm 8.6)\cdot 10^{-1}$	$(-1.7\pm 4.4)\cdot 10^{-1}$	$(2.4\pm 7.0)\cdot 10^{-1}$	<0.05	<0.05	<0.05
	Niemierko	$(-4.0\pm 8.5)\cdot 10^{-1}$	$(-1.7\pm 4.4)\cdot 10^{-1}$	$(2.3\pm 7.0)\cdot 10^{-1}$	<0.05	<0.05	<0.05
		*Prostate Bed*					
King	Poisson	−0.3±1.6	$(-3.5\pm 7.2)\cdot 10^{-1}$	$(0.3\pm 8.0)\cdot 10^{-1}$	0.73	0.23	<0.05
	Sigmoidal	−0.3±1.5	$(-4.3\pm 8.0)\cdot 10^{-1}$	$(0.5\pm 8.2)\cdot 10^{-1}$	0.65	0.09	<0.05
	Niemierko	−0.3±1.5	$(-4.4\pm 7.7)\cdot 10^{-1}$	$(0.4\pm 8.1)\cdot 10^{-1}$	0.68	0.10	<0.05
Okunieff	Poisson	$(-0.2\pm 1.5)\cdot 10^{-2}$	$(-3.1\pm 6.5)\cdot 10^{-3}$	$(0.3\pm 7.3)\cdot 10^{-3}$	0.71	0.24	<0.05
	Sigmoidal	$(-0.9\pm 5.2)\cdot 10^{-1}$	$(-1.3\pm 3.5)\cdot 10^{-1}$	$(0.4\pm 3.3)\cdot 10^{-1}$	0.62	0.08	<0.05
	Niemierko	$(-0.9\pm 3.8)\cdot 10^{-1}$	$(-1.1\pm 2.0)\cdot 10^{-1}$	$(0.1\pm 2.0)\cdot 10^{-1}$	0.70	0.09	<0.05
Levegrun	Poisson	−0.6±3.4	−0.8±1.5	0.1±1.7	0.75	0.21	<0.05
	Sigmoidal	−0.5±2.0	−0.6±1.1	0.1±1.1	0.66	0.09	<0.05
	Niemierko	−0.5±2.1	−0.6±1.1	0.1±1.1	0.69	0.09	<0.05

M3D=Mobius3D; CC=COMPASS dose calculation; CR=COMPASS dose reconstruction.

**Table 10:** Calculations and comparisons of NTCP values for 100 analyzed prostate treatments, 25 from each group (high‐, intermediate‐, low‐risk, and prostate bed patients), using the two models described in the text

		Mean Differences (%)			p‐values		
*Volume*	*Model*	*M3D*	*CC*	*CR*	*M3D vs. CC*	*M3D vs. CR*	*CC vs. CR*
		*High‐risk Prostate*					
Rectum	LKB	$(8.7\pm 8.7)\cdot 10^{-1}$	$(0.3\pm 2.6)\cdot 10^{-1}$	$(9.1\pm 5.0)\cdot 10^{-1}$	<0.05	0.68	<0.05
	Niemierko	$(2.4\pm 3.5)\cdot 10^{-1}$	$(0.5\pm 8.9)\cdot 10^{-2}$	$(2.4\pm 2.2)\cdot 10^{-1}$	<0.05	0.97	<0.05
Bladder	LKB	$(-3.7\pm 5.2)\cdot 10^{-2}$	$(0.9\pm 1.4)\cdot 10^{-2}$	$(-2.8\pm 3.2)\cdot 10^{-2}$	<0.05	0.49	<0.05
	Niemierko	$(-3.4\pm 4.2)\cdot 10^{-2}$	$(0.9\pm 1.3)\cdot 10^{-2}$	$(-2.7\pm 3.0)\cdot 10^{-2}$	<0.05	0.55	<0.05
LF head	LKB	$(0.6\pm 2.0)\cdot 10^{-4}$	$(0.9\pm 2.5)\cdot 10^{-4}$	$(3.7\pm 8.2)\cdot 10^{-5}$	0.06	0.48	0.23
	Niemierko	$(0.6\pm 1.7)\cdot 10^{-4}$	$(0.9\pm 2.2)\cdot 10^{-4}$	$(4.1\pm 7.6)\cdot 10^{-5}$	<0.05	0.54	0.22
RF head	LKB	$(1.5\pm 6.1)\cdot 10^{-4}$	$(1.3\pm 4.0)\cdot 10^{-4}$	$(1.0\pm 2.4)\cdot 10^{-4}$	0.65	0.55	0.45
	Niemierko	$(1.3\pm 4.8)\cdot 10^{-4}$	$(1.2\pm 3.0)\cdot 10^{-4}$	$(1.0\pm 1.9)\cdot 10^{-4}$	0.76	0.64	0.50
		*Intermediate‐risk Prostate*					
Rectum	LKB	$(8.6\pm 5.1)\cdot 10^{-1}$	$(0.9\pm 2.0)\cdot 10^{-1}$	1.2±0.6	<0.05	<0.05	<0.05
	Niemierko	$(2.2\pm 1.9)\cdot 10^{-1}$	$(2.4\pm 5.8)\cdot 10^{-2}$	$(3.1\pm 2.3)\cdot 10^{-1}$	<0.05	<0.05	<0.05
Bladder	LKB	$(-0.1\pm 1.5)\cdot 10^{-3}$	$(0.4\pm 1.5)\cdot 10^{-3}$	$(-1.2\pm 3.2)\cdot 10^{-3}$	<0.05	0.14	0.08
	Niemierko	$(-0.2\pm 2.1)\cdot 10^{-3}$	$(0.7\pm 1.9)\cdot 10^{-3}$	$(-1.9\pm 4.1)\cdot 10^{-3}$	<0.05	0.08	<0.05
LF head	LKB	$(2.0\pm 6.2)\cdot 10^{-6}$	$(2.6\pm 7.2)\cdot 10^{-6}$	$(3.4\pm 8.6)\cdot 10^{-6}$	<0.05	<0.05	0.05
	Niemierko	$(2.7\pm 8.1)\cdot 10^{-6}$	$(3.6\pm 9.4)\cdot 10^{-6}$	$(0.5\pm 1.1)\cdot 10^{-5}$	<0.05	<0.05	<0.05
RF head	LKB	$(2.9\pm 6.3)\cdot 10^{-6}$	$(0.5\pm 1.2)\cdot 10^{-5}$	$(0.8\pm 2.3)\cdot 10^{-5}$	0.15	0.14	0.14
	Niemierko	$(3.7\pm 7.8)\cdot 10^{-6}$	$(0.6\pm 1.4)\cdot 10^{-5}$	$(1.0\pm 2.7)\cdot 10^{-5}$	0.12	0.12	0.12
		*Low‐risk Prostate*					
Rectum	LKB	1.1±0.7	$(1.5\pm 2.9)\cdot 10^{-1}$	1.5±0.5	<0.05	<0.05	<0.05
	Niemierko	$(3.1\pm 3.6)\cdot 10^{-1}$	$(0.5\pm 1.2)\cdot 10^{-1}$	$(4.0\pm 3.1)\cdot 10^{-1}$	<0.05	<0.05	<0.05
Bladder	LKB	$(1.5\pm 7.1)\cdot 10^{-3}$	$(1.6\pm 5.7)\cdot 10^{-3}$	$(-2.0\pm 5.5)\cdot 10^{-3}$	0.87	0.15	0.11
	Niemierko	$(1.7\pm 8.2)\cdot 10^{-3}$	$(2.1\pm 6.4)\cdot 10^{-3}$	$(-2.7\pm 6.3)\cdot 10^{-3}$	0.41	0.12	0.06
LF head	LKB	$(0.3\pm 1.1)\cdot 10^{-5}$	$(2.5\pm 9.1)\cdot 10^{-6}$	$(0.3\pm 1.0)\cdot 10^{-5}$	0.71	0.49	0.12
	Niemierko	$(0.3\pm 1.3)\cdot 10^{-5}$	$(0.3\pm 1.1)\cdot 10^{-5}$	$(0.4\pm 1.2)\cdot 10^{-5}$	0.85	0.35	0.12
RF head	LKB	$(1.9\pm 8.5)\cdot 10^{-6}$	$(2.0\pm 6.9)\cdot 10^{-6}$	$(0.3\pm 1.3)\cdot 10^{-5}$	0.88	0.09	0.23
	Niemierko	$(0.2\pm 1.0)\cdot 10^{-5}$	$(2.6\pm 8.5)\cdot 10^{-6}$	$(0.4\pm 1.6)\cdot 10^{-5}$	0.70	0.07	0.21
		*Prostate Bed*					
Rectum	LKB	1.1±0.6	$(1.1\pm 3.8)\cdot 10^{-1}$	1.3±0.6	<0.05	<0.05	<0.05
	Niemierko	$(2.9\pm 1.8)\cdot 10^{-1}$	$(0.3\pm 1.2)\cdot 10^{-1}$	$(3.6\pm 2.1)\cdot 10^{-1}$	<0.05	<0.05	<0.05
Bladder	LKB	$(-3.3\pm 7.5)\cdot 10^{-3}$	$(1.8\pm 5.1)\cdot 10^{-3}$	$(-0.4\pm 1.1)\cdot 10^{-2}$	<0.05	0.76	<0.05
	Niemierko	$(-4.2\pm 8.3)\cdot 10^{-3}$	$(2.2\pm 5.5)\cdot 10^{-3}$	$(-0.5\pm 1.2)\cdot 10^{-2}$	<0.05	0.71	<0.05
LF head	LKB	$(0.6\pm 2.3)\cdot 10^{-5}$	$(1.0\pm 2.7)\cdot 10^{-5}$	$(1.5\pm 4.7)\cdot 10^{-5}$	<0.05	0.08	0.22
	Niemierko	$(0.7\pm 2.3)\cdot 10^{-5}$	$(1.1\pm 2.7)\cdot 10^{-5}$	$(1.7\pm 4.8)\cdot 10^{-5}$	<0.05	<0.05	0.19
RF head	LKB	$(1.6\pm 3.1)\cdot 10^{-6}$	$(0.6\pm 1.8)\cdot 10^{-5}$	$(5.0\pm 7.2)\cdot 10^{-6}$	0.20	<0.05	0.65
	Niemierko	$(2.3\pm 4.3)\cdot 10^{-6}$	$(0.8\pm 1.9)\cdot 10^{-5}$	$(7.0\pm 9.2)\cdot 10^{-6}$	0.17	<0.05	0.80

M3D=Mobius3D; CC=COMPASS dose calculation; CR=COMPASS dose reconstruction.

The correlation between the changes in DVH parameters and the corresponding changes in radiobiological outcomes is summarized in Tables 11 and 12 for targets and normal tissues, respectively. Selected DVH parameters for prostate volumes were $D_{98},D_{2}$, and $D_{50}$. Correlation ranged from moderate (r>0.500) to strong (r>0.700) for gEUD comparisons, although CC results seemed to have a weaker correlation (r<0.500). For OARs, selected DVH parameters were $V_{70}$ and mean dose for rectum and bladder and maximum dose for femoral heads. For gEUD, moderate to strong correlation was observed, although some results exhibited a weaker correlation (CC and CR results for rectum and bladder). NTCP results seemed to have stronger correlation for the LKB model than the Niemierko model for rectum. Correlation was weaker for mean dose compared to $V_{70}$ values for bladder. Low‐risk cases exhibited a stronger correlation for bladder NTCP results compared with the other cases. Femoral heads showed no correlation for either LKB or Niemierko calculations.

**Table 11:** Pearson correlation coefficient comparing gEUD and TCP for prostate target volumes. The selected DVH metrics were $D_{98},D_{2}$, and $D_{50}$

			*Cheung/King Parameters*			*Okunieff Parameters*			*Levegrun Parameters*		
		*gEUD*	*TCP Poiss*	*TCP Sigm*	*TCP Niem*	*TCP Poiss*	*TCP Sigm*	*TCP Niem*	*TCP Poiss*	*TCP Sigm*	*TCP Niem*
		*High‐risk Prostate*									
$D_{98}$	M3D	0.582	0.599	0.527	0.581	0.593	0.516	0.581	0.595	0.579	0.579
	CC	0.335	0.396	0.254	0.336	0.393	0.242	0.337	0.394	0.338	0.340
	CR	0.339	0.469	0.242	0.344	0.471	0.227	0.346	0.470	0.353	0.356
$D_{2}$	M3D	0.847	0.721	0.799	0.841	0.704	0.786	0.838	0.709	0.823	0.818
	CC	0.368	0.063	0.348	0.358	0.046	0.337	0.354	0.051	0.340	0.327
	CR	0.752	0.630	0.650	0.747	0.614	0.627	0.745	0.619	0.732	0.727
$D_{50}$	M3D	0.917	0.889	0.863	0.916	0.879	0.852	0.916	0.882	0.913	0.912
	CC	0.783	0.886	0.645	0.786	0.880	0.624	0.787	0.882	0.788	0.793
	CR	0.883	0.835	0.736	0.880	0.817	0.708	0.880	0.823	0.869	0.868
		*Intermediate‐risk Prostate*									
$D_{98}$	M3D	0.593	0.596	0.574	0.590	0.596	0.529	0.591	0.597	0.589	0.589
	CC	0.476	0.664	0.389	0.486	0.662	0.211	0.481	0.667	0.480	0.489
	CR	0.246	0.463	0.220	0.276	0.464	0.074	0.260	0.461	0.280	0.287
$D_{2}$	M3D	0.749	0.752	0.694	0.734	0.750	0.593	0.741	0.753	0.731	0.729
	CC	0.409	0.370	0.352	0.406	0.367	0.230	0.408	0.373	0.405	0.404
	CR	0.662	0.651	0.582	0.655	0.648	0.364	0.659	0.655	0.653	0.652
$D_{50}$	M3D	0.846	0.877	0.811	0.843	0.877	0.724	0.845	0.878	0.842	0.842
	CC	0.641	0.863	0.536	0.656	0.860	0.308	0.648	0.866	0.652	0.661
	CR	0.764	0.934	0.671	0.776	0.932	0.382	0.770	0.935	0.775	0.780
		*Low‐risk Prostate*									
$D_{98}$	M3D	0.559	0.585	0.466	0.555	0.585	0.435	0.556	0.590	0.547	0.549
	CC	0.415	0.541	0.337	0.415	0.541	0.315	0.416	0.541	0.409	0.414
	CR	0.549	0.646	0.464	0.556	0.646	0.425	0.554	0.646	0.558	0.562
$D_{2}$	M3D	0.701	0.691	0.626	0.700	0.691	0.596	0.700	0.694	0.698	0.697
	CC	0.402	0.539	0.281	0.397	0.539	0.249	0.398	0.539	0.389	0.392
	CR	0.700	0.758	0.590	0.703	0.758	0.540	0.702	0.758	0.703	0.705
$D_{50}$	M3D	0.853	0.858	0.756	0.851	0.857	0.719	0.852	0.862	0.846	0.846
	CC	0.530	0.882	0.320	0.531	0.882	0.262	0.531	0.882	0.525	0.532
	CR	0.856	0.956	0.711	0.860	0.955	0.648	0.859	0.957	0.861	0.864
		*Prostate Bed*									
$D_{98}$	M3D	0.848	0.885	0.833	0.850	0.883	0.740	0.849	0.888	0.840	0.850
	CC	0.484	0.563	0.425	0.472	0.556	0.260	0.479	0.573	0.447	0.475
	CR	0.535	0.614	0.525	0.543	0.616	0.441	0.539	0.610	0.531	0.541
$D_{2}$	M3D	0.660	0.655	0.634	0.652	0.650	0.533	0.657	0.663	0.644	0.654
	CC	0.382	0.465	0.340	0.380	0.460	0.188	0.382	0.472	0.358	0.381
	CR	0.591	0.706	0.544	0.595	0.701	0.316	0.593	0.712	0.567	0.594
$D_{50}$	M3D	0.926	0.944	0.907	0.925	0.941	0.806	0.926	0.949	0.915	0.926
	CC	0.715	0.876	0.654	0.718	0.870	0.424	0.717	0.882	0.682	0.718
	CR	0.796	0.900	0.751	0.800	0.895	0.526	0.798	0.904	0.773	0.799

M3D=Mobius3D; CC=COMPASS dose calculation; CR=COMPASS dose reconstruction; Poiss=Poisson; Sigm=sigmoidal; Niem=Niemierko.

**Table 12:** Pearson correlation coefficient comparing gEUD and NTCP for OARs. The selected DVH metrics were $V_{70}$ and mean dose ($D_{M}$) for rectum and bladder, and maximum dose ($D_{\text{max}}$) for femoral heads

		*High‐risk Prostate*			*Intermediate‐risk Prostate*			*Low‐risk Prostate*			*Prostate Bed*		
		*gEUD*	*NTCP LKB*	*NTCP Niem*	*gEUD*	*NTCP LKB*	*NTCP Niem*	*gEUD*	*NTCP LKB*	*NTCP Niem*	*gEUD*	*NTCP LKB*	*NTCP Niem*
		*Rectum*											
$V_{70}$	M3D	0.820	0.944	0.847	0.603	0.893	0.795	0.502	0.889	0.766	0.839	0.938	0.804
	CC	0.769	0.788	0.741	0.655	0.733	0.720	0.418	0.489	0.470	0.826	0.820	0.781
	CR	0.397	0.915	0.796	0.165	0.893	0.866	0.017	0.881	0.784	0.680	0.906	0.823
$D_{M}$	M3D	0.881	0.859	0.729	0.777	0.564	0.351	0.506	0.775	0.608	0.743	0.736	0.600
	CC	0.729	0.603	0.514	0.765	0.653	0.504	0.778	0.575	0.344	0.578	0.493	0.438
	CR	0.706	0.666	0.470	0.672	0.492	0.335	0.707	0.269	0.132	0.620	0.189	0.002
		*Bladder*											
$V_{70}$	M3D	0.777	0.639	0.662	0.643	0.206	0.266	0.748	0.815	0.816	0.594	0.616	0.594
	CC	0.163	0.142	0.162	0.114	0.160	0.189	0.196	0.578	0.607	0.445	0.097	0.445
	CR	0.735	0.641	0.668	0.505	0.379	0.48	0.587	0.518	0.581	0.719	0.542	0.719
$D_{M}$	M3D	0.853	0.462	0.497	0.522	0.130	0.143	0.820	0.452	0.455	0.664	‐0.16	‐0.153
	CC	0.449	0.158	0.210	0.424	−0.306	−0.335	0.416	−0.342	−0.374	0.397	−0.098	−0.072
	CR	0.825	0.557	0.594	0.748	−0.027	0.056	0.751	0.298	0.336	0.723	0.389	0.418
		*Left Femoral Head*											
$D_{\text{max}}$	M3D	0.847	0.104	0.115	0.418	0.351	0.350	0.693	0.190	0.190	0.689	−0.171	−0.155
	CC	0.675	0.022	0.030	0.421	0.483	0.478	0.432	−0.085	−0.084	0.704	−0.184	−0.167
	CR	0.851	−0.338	−0.288	0.319	0.285	0.280	0.844	−0.122	−0.120	0.637	−0.309	−0.311
		*Right Femoral Head*											
$D_{\text{max}}$	M3D	0.901	0.067	0.091	0.552	0.139	0.190	0.496	0.077	0.078	0.745	0.183	0.203
	CC	0.705	0.066	0.072	0.589	−0.048	−0.017	0.376	−0.198	−0.181	0.524	0.057	0.067
	CR	0.815	0.049	0.070	0.531	−0.160	−0.143	0.507	−0.174	−0.176	0.723	−0.313	−0.287

M3D=Mobius3D; CC=COMPASS dose calculation; CR=COMPASS dose reconstruction; Niem=Niemierko.

## IV. DISCUSSION

### A. Remarks and limitations of the present study

#### A.1 Variability between radiobiological response parameters and models

Radiobiological models are powerful evaluation tools because values such as TCP and NTCP are related to the clinical outcome of a treatment. Therefore, biological‐based evaluation becomes an interesting metric in order to evaluate a treatment plan. However, all biological models have uncertainties in the values of the parameters chosen. These metrics should be used with caution, due to these uncertainties. The use of biological models in plan evaluation requires accurate TCP/NTCP models and parameter estimation. The users of biological metrics could derive model parameters based on their own experience by calibrating selected models against observed clinical outcomes. Another option is to cautiously use published parameter values, as these data are available for many tumor and normal tissue sites. These known ideas have been expressed in reference reports, such as AAPM TG‐166 or ICRU 83 reports.^20^, ^21^ The first option could not be feasible for our institution, as it requires expertise in outcome modeling and sufficient patient throughput. In order to overcome these possible uncertainties, published parameters taken from reference reports and studies were used, as in the TG‐166 report,^(21)^ the study by Okunieff et al.,^(45)^ or the study by Burman et al.^(58)^ The possible variation due to the model selection was overcome by using three models for TCP calculations (Poisson, sigmoidal, and Niemierko) and two models for NTCP calculations (LKB and Niemierko). In both cases, results between the models were comparable.

A limitation of the present study was related with the TCP calculation using the Eq. (3), where the dose protraction factor has not been considered in order to model the TCP. Dose protraction factor (G) modifies the quadratic term of the linear‐quadratic expression in order to take into account the sublethal damage repair of protracting the dose delivery. If the delivery takes a short time (instantaneous), G=1. For any other dose delivery pattern, G<1. This study was performed considering G ~ 1 since treatment times are about few minutes, as in the prostate treatments using VMAT techniques.

#### A.2 Use of AAPM TG‐166 test cases

The aim of the present study was to evaluate the implementation of radiobiological metrics in the patient‐specific QA workflow. TG‐166 test cases have been designed to assess the biological modeling implemented in several commercial TPSs. In particular, benchmark phantom structures have been specifically developed to address this problem. In addition, the dose calculation and reconstruction capabilities of the systems should be tested with clinical cases. The other TG‐166 cases (H&N, prostate, and brain) have been intentionally used for this purpose.

#### A.3 Relevance of radiobiological metrics

The main aim of patient‐specific QA for modulated treatments is to ensure the quality of each individual patient treatment. The pretreatment QA measurement‐based process must be considered to ensure the correct information flow from TPS plan calculation to treatment delivery in the linac by means of the record and verify (R&V) system. Such patient‐specific QA is conventionally performed by delivering the patient plans to a phantom with detectors, and comparing the calculated and measured dose in the phantom. Recent studies have investigated estimating the delivered patient dose from QA measurement, resulting in several new QA tools. These new approaches to patient‐specific QA open the possibility of adopting patient dose‐based metrics that are more relevant to the expected treatment outcome. Therefore, new QA metrics should be introduced in order to effectively take into account the clinical impact of possible calculation/delivery mistakes on the treatment outcome for modulated treatments. Considering radiobiological parameters as potential indicators of this clinical outcome for radiation therapy treatments, such parameters could be included in these metrics. The previous statement goes beyond the usual patient‐specific QA flow, which is based in dose distribution (2D or 3D) comparisons. In this way, patient‐specific QA metrics could directly evaluate the variation of expected clinical outcome between the planned dose and delivered dose.

In addition to the previous discussion, radiobiological metrics could be used to evaluate the robustness of a radiotherapy treatment from the point of view of its sensitivity to possible perturbations. From the sigmoid shape of TCP and NTCP curves, the high region for TCP and the low region for NTCP are the ideal regions for these indices, because results are less sensitive to possible changes. Determining the robustness of a plan could be used to prevent the influence of different possible sources of error and could lead to improvements in the quality of the plan before treatment.

#### A.4 Limitation in the definition of action levels

Any QA metric should have sensitivity in order to show the impact of possible errors on the patient treatment. Action levels for classical metrics, as gamma analysis, have been widely studied. However, gamma passing rates could not catch clinically relevant patient dose errors.^16^, ^17^ TCP and NTCP models have been introduced in order to take into account the treatment outcome. In this way, these metrics permit to concentrate on the errors that are of clinical importance. However, the impact of an error source on the clinical outcome depends on the location/characteristics of the PTV and the possible OARs. As an example, the traditional action levels for gamma analysis (3% of dose difference) could be applied to evaluate the observed difference in a serial normal tissue, as the spinal cord. If the maximum dose to spinal cord is, for example, 5 Gy, an error of 3%, 5% or even 10% probably will have no impact on the NTCP for this tissue. When the NTCP slope starts to be steep, the possible impact on NTCP becomes increasingly important. In order to implement new patient‐specific QA protocols, action levels for new metrics should be defined. Nevertheless, the definition of action levels might be beyond the scope of the present study. Future application of these metrics to a large amount of modulated treatments/disease sites/PTVs/OARs could give us enough information and statistics in order to define a correct level for the acceptability/rejection of gEUD/TCP/ NTCP variations, depending on the case.

### B. Verifications of biological metrics for both systems with TG‐166

Discrepancies between TPS and the two systems were in good agreement for gEUD calculations. Maximum discrepancies were found for small volume structures, like inner ear, where small differences could lead to poor results. For TCP and NTCP comparisons, discrepancies were better than the previous results. The worst results were found in the benchmark phantom test case.

### C. Evaluation of prostate treatments with classical (gamma) and DVH‐based dose‐volume metrics

COMPASS‐calculated and reconstructed passing rates were slightly better than those from Mobius3D. Results were above the TG‐119 action level of 88% for composite dose gamma analysis,^(62)^ excluding some results with the 2%/2mm gamma criterion: COMPASS calculated mean values for high‐risk pelvic lymph node PTV, COMPASS reconstructed mean values for high‐risk prostate PTV, and some Mobius3D results (high‐risk prostate and pelvic lymph node PTVs, prostate bed PTV, rectum for the bed case, and bladder for the high‐ and low‐risk and bed cases).

DVH comparisons were comparable to results from other studies.^63^, ^64^, ^65^ Both systems led to similar results for dose‐volume parameters. CC calculations improved the M3D values, and Mobius3D results were better than CR values.

### D. Biological metrics applied prostate treatments

Radiobiological calculations led to comparable discrepancies for the three systems. The statistically significant differences were shown in the previous section. For gEUD values, differences were larger for OARs than for target volumes. Considering the TCP calculations, absolute TCP values calculated for the TPS with the Poisson model were, in general, slightly larger than those from the sigmoidal and Niemierko models. These absolute values were comparable between the models, with the exception of the results for the high‐risk prostate case taking the values from Cheung et al.^(54)^ (sigmoidal and Niemierko values were very close, but they were about 20% lower than Poisson results), and the prostate bed case taking the values from King et al.^(56)^ (sigmoidal and Niemierko values were also very close but about 15% lower than Poisson results) and Levegrun et al.^(57)^ (Poisson results were about 10%‐15% higher than those from sigmoidal and Niemierko values.) The observed discrepancies in absolute TCP values come from the model/parameter selection and they were also derived for M3D, CC, and CR results, preserving the low differences obtained in the comparisons. For NTCP calculations, absolute values were comparable between both the LKB and Niemierko models and also preserved the low discrepancies in the comparisons.

**Figure 4:**
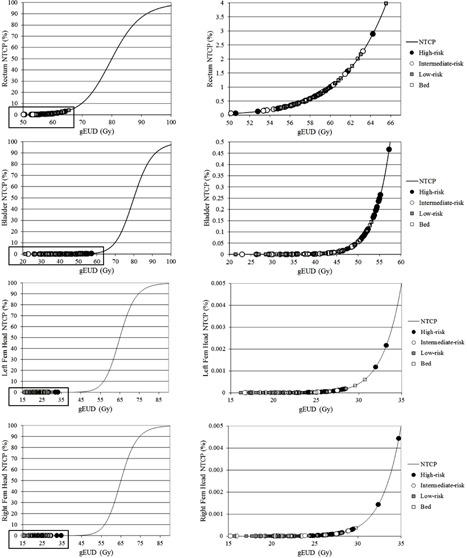
NTCP values for rectum, bladder, and femoral heads calculated by the TPS for the high‐, intermediate‐, and low‐risk and prostate bed cases. NTCP values are located in low‐gradient regions of the sigmoid curve; therefore, the clinical outcome is less sensitive to changes in DVH parameters.

Correlation results between DVH and gEUD/TCP/NTCP differences were analyzed in the previous section. Correlation between $D_{50}$ and TCP was stronger than results obtained comparing $D_{98}$ and $D_{2}$. TCP increase/decrease was highly correlated with $D_{50}$, which is related to the mean dose. Correlation between NTCP and dose‐volume parameters for OARs was larger for rectum than bladder or femoral head values. These results could be explained using ideas from the study by Zhen et al.,^(66)^ based on the discussion expressed in Discussion section A.3 above. If TCP/NTCP values are located in low‐gradient regions of the sigmoid curve (high TCP or low NTCP), the clinical outcome is less sensitive to changes in the plan, like dose‐volume discrepancies. NTCP values for bladder and femoral heads are low and extremely low, respectively (Fig. 4). This could explain the lack of correlation between changes in DVH parameters and NTCP results in these cases.

## V. CONCLUSIONS

This study has explored the potential usefulness of biological metrics in patient‐specific QA process. Initial evaluation of radiobiological data extracted from dose calculation and reconstruction performed by Mobius3D and COMPASS systems were carried out by means of TG‐166 test cases. The capabilities of the systems for 3D dose calculations and reconstructions were assessed with classical metrics, obtaining comparable results between both systems. Radiobiological metrics expressed in terms of comparisons between different indices (gEUD, TCP, and NTCP) were applied to a paradigmatic case of VMAT delivery (prostate treatment). The possibility of using radiobiological calculations as alternative metrics was introduced in order to evaluate the expected clinical outcome of radiotherapy treatments in the scope of pretreatment patient‐specific QA.

## COPYRIGHT

This work is licensed under a Creative Commons Attribution 4.0 International License.
